# Linking patient-specific basal MET phosphorylation levels to liver health

**DOI:** 10.1038/s44320-024-00023-y

**Published:** 2024-02-16

**Authors:** Fabian Fröhlich

**Affiliations:** Dynamics of Living Systems Laboratory, The Francis Crick Institute, London, UK

**Keywords:** Molecular Biology of Disease, Proteomics, Signal Transduction

## Abstract

Integrative systems medicine approaches can help predicting clinical outcomes. In their recent study, Klingmüller and colleagues (Burbano de Lara et al, 2024) integrate proteomic data with dynamic pathway modelling to infer patient-specific parameters that predict patient outcomes after liver surgery.

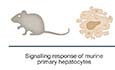

## Teaser

Systems medicine aims at enhancing patient outcomes by integrating molecular profiles and computational methods. In their recent study, Klingmüller and colleagues (Burbano de Lara et al, 2024) integrated proteomic data with mathematical models of signal transduction to infer patient-specific parameters that predict patient outcomes following liver surgery.

**Figure 1:**
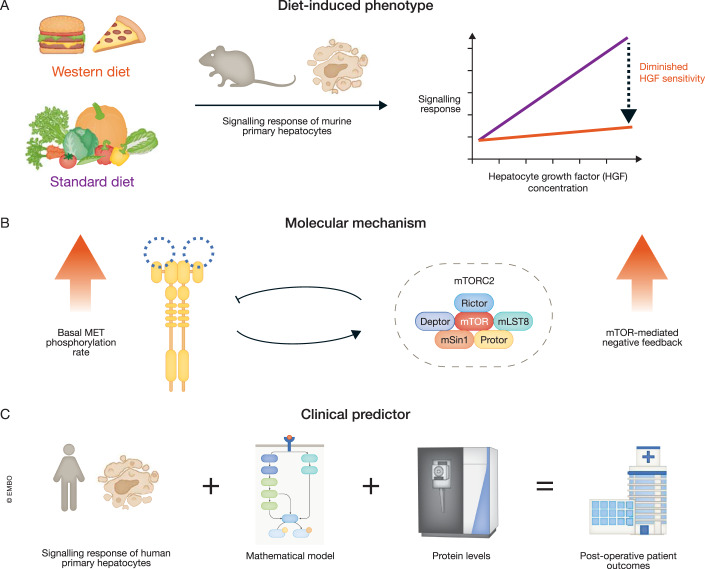
From preclinical phenotypes to clinical predictors. (**A**) Western diet promotes a diminished sensitivity of PI3K signalling to stimulation by hepatocyte growth factor (HGF). (**B**) Diminished HGF sensitivity is explained by the activation of a mammalian target of rapamycin (mTOR)-mediated negative feedback, which is in turn activated by an increased basal MET phosphorylation rate. (**C**) Integrating signalling response data from primary human hepatocytes with a mathematical pathway model and protein levels measured by mass spectrometry identifies basal MET phosphorylation as a predictor of post-operative patient outcomes.

The incidence of the metabolic dysfunction-associated steatotic liver (MASLD), formerly known as non-alcoholic fatty liver disease (NALFD), has increased in the last few decades. If sustained, MASLD can lead to liver damage and even liver cancer. It is therefore important to understand the mechanisms of MASLD development and progression. MASLD has been linked to high-sugar and high-fat Western diets (Romero-Gómez et al, 2017). Considering this association, Burbano de Lara et al, 2024 started their mechanistic exploration of MASLD by characterising the proteomic changes induced by a Western diet in a preclinical mouse model. This revealed alterations in metabolic and signal transduction pathways regulating cell proliferation. The authors then quantified diet-induced changes in the dynamics of two major mammalian signalling pathways governing cell proliferation, namely phosphoinositide 3-kinase (PI3K) and mitogen-activated protein kinase (MAPK) signalling. These pathways can be activated by the hepatocyte growth factor (HGF), the ligand for receptor tyrosine kinase MET, which is a central player in liver development, pathology and repair (Nakamura and Mizuno, 2010). Experiments in murine primary hepatocytes, the predominant cell type in the liver, indicated that a Western diet reduced the responsiveness of PI3K signalling to HGF (Fig. 1A).

To better understand the molecular mechanisms contributing to the reduced HGF sensitivity, the authors developed a mathematical model of PI3K and MAPK signalling to integrate HGF signalling responses with proteomic data. The levels of core PI3K and MAPK pathway proteins were measured in individual mice using Data Independent Acquisition (DIA) mass spectrometry, a technique well-suited for quantifying low-abundance signalling proteins (Doerr, 2015). The mathematical model allowed them to demonstrate that the variability in HGF signalling responses observed across primary hepatocytes from distinct mice could be attributed to both mouse-specific differences in protein levels as well as a single, diet-dependent model parameter: the basal (HGF-independent) phosphorylation rate of the MET receptor. Analyses using the model indicated that elevated basal MET phosphorylation rates induced by a Western diet activated the mammalian target of rapamycin (mTOR)-mediated negative feedback on PI3K signalling, explaining the reduced sensitivity (Fig. 1B).

To assess if their findings from mouse models are relevant for human biology, the authors also measured protein levels and HGF signalling responses in patient-derived primary hepatocytes and adapted the mouse model to human. They inferred patient-specific values for the basal MET receptor phosphorylation rate and observed higher levels of phosphorylated MET hepatocytes from patients with high degree of steatosis. Remarkably, the model successfully explained organism- and individual-specific variations in HGF signalling responses and only required the introduction of two additional human-specific model parameters: the HGF-induced MET phosphorylation rate and the degradation rate of phosphorylated MET receptors. Subsequently, the authors conducted a comparative analysis of patient outcomes following liver surgery, incorporating three types of patient-specific metrics: model parameters, predictions of MAPK and PI3K signalling responses to HGF stimulation, and experimentally measured ex-vivo HGF-induced proliferation scores. Only the inferred patient-specific basal MET receptor phosphorylation rate served as a good predictor for patient outcomes (Fig. 1C).

This study marks a notable achievement in bridging the gap between preclinical mouse models and human biology and builds on a previous key finding of the authors’ lab (Adlung et al, 2017), namely that differences in the basal protein abundance of certain pathway components affect the dynamics of information processing. The accomplishment is rooted in two key factors. First, the study highlights that most of the variability between individuals of an organism as well as across organisms can be explained using the difference in protein levels: only three organism-, patient-, and/or diet-specific parameters needed to be introduced in the model. Variability in signalling response seems to be primarily generated at the receptor level, with the help of negative feedbacks, consistent with previous studies of MAPK signalling (Shi et al, 2016). Second, the authors employed a mathematical model grounded in the biochemical laws that govern signalling dynamics universally across both organisms. Despite simplifications, the model appears to maintain the fundamental nature of these biochemical laws.

Nevertheless, model parameters should not be interpreted as fundamental biochemical constants. For example, the basal MET phosphorylation rate is a phenomenological description of the net effect of unknown molecular mechanisms. The authors hypothesize that these mechanisms could be related to membrane composition or crosstalk with insulin signalling. The phenomenological nature of this parameter justifies the assumption that it may be diet- or patient-specific. However, the phenomenological nature also potentially hampers its direct clinical application, as it seems impractical to generate for every patient the dynamic response data from patient-derived primary cells that would be required to infer this parameter value. In this study, the authors avoid this issue by proposing baseline MET phosphorylation levels as a clinically available proxy for basal MET phosphorylation rates. Still, broader applicability of the approach will either require more mechanistically detailed models that can extract the source of all variability from proteomic data or the integration with machine-learning methods directed at predicting the values of phenomenological parameters from molecular profiles.
